# Empowering Parent Carers of Children With Neurodisability in Healthcare Settings: Co‐Producing a Theory of Change for Intervention Development

**DOI:** 10.1111/hex.70871

**Published:** 2026-09-09

**Authors:** Jim Reeder, Saira Minhas, Erica Breuer, Jane R. Smith, Sally Kendall, Christopher Morris

**Affiliations:** ^1^ PenCRU (Peninsula Childhood Disability Research Unit), Health & Community Sciences University of Exeter Medical School UK; ^2^ PenCRU Family Faculty, Health & Community Sciences University of Exeter Medical School UK; ^3^ Department of Medicine and Public Health The University of Newcastle Newcastle New South Wales Australia; ^4^ Exeter Collaboration for Academic Primary Care (APEx), University of Exeter Medical School University of Exeter UK; ^5^ Centre for Health Services Studies University of Kent UK

## Abstract

**Background:**

Parent carers of children with neurodisability are empowered when they have greater agency and influence over decisions about their child's care. When parent carers are empowered, it benefits both them and their children. Building on previous work, this study aimed to co‐produce a Theory of Change (ToC) to inform the development of a new parent carer empowerment intervention.

**Methods:**

We convened a group of nine participants (four parent carers, three service providers, one service manager, one service commissioner) to co‐produce our ToC. The participatory process consisted of structured workshops co‐facilitated by a researcher and a parent carer partner, with iterative phases of data analysis. Analysis drew on behavioural science to develop detailed ToC maps and a provisional intervention plan.

**Findings:**

Twenty‐nine short‐term, thirteen medium‐term and seven long‐term outcomes were identified and incorporated into causal pathways of the ToC. The group prioritised two medium‐term outcomes informing the focus of the intervention: (1) Effective two‐way information sharing and (2) trusting relationships between service providers and parent carers. Following integration of a behavioural analysis, the new intervention targets change in seven service provider behaviours.

**Conclusions:**

Our co‐produced, theory and evidenced‐based intervention targets change in service provider behaviour by focusing on self‐awareness and critical reflection, specifically related to how information is shared/managed and how interactions with parent carers are framed and performed.

**Patient/Public Contribution:**

Our project team includes a group of parent carer research partners. Members of the group have been involved in planning and design, facilitation of workshops and dissemination of findings.

## 1 Introduction

Neurodisability has been defined as ‘a group of congenital or acquired long‐term conditions that are attributed to impairment of the brain and/or neuromuscular system and create functional limitations’ [1]. This includes ‘developmental’ diagnoses, such as autism, attention deficit hyperactivity disorder and intellectual disability; as well as ‘medical’ diagnoses, such as cerebral palsy, epilepsy and Down's syndrome [2]. In the UK, the prevalence of childhood disability is estimated at 7.3% [3], and children with neurodisability make up the majority of this group [4].

In UK legislation, a parent carer is defined as ‘any person aged 18 or over who provides or intends to provide care for a disabled child for whom the person has parental responsibility’ [5]. For children with neurodisability, parent carers play a key advocacy role as they seek to communicate with service providers and navigate often complex health, education and social care systems. This responsibility, along with other magnified caregiving demands, can carry unique challenges for parent carers and can have a significant impact on their financial, emotional, physical and social wellbeing [6].

Based on the World Health Organisation definition for patient empowerment [7], in our previous work we have described parent carer empowerment as the strengthening of parental control and influence over the decisions and actions related to their child's care [8]. Benefits of parent carer empowerment include improved parent carer well‐being [9], reduced reliance on health professionals [10], improved cost‐effectiveness of services [11] and improved health outcomes for children [12]. These recognised benefits are reflected in contemporary health and social care policy in the UK, which increasingly calls for service users to be supported to become active participants in their own care [5, 13, 14, 15].

Over the past 10 years, there has been a proliferation of initiatives to support different aspects of parent carer empowerment. Our recently published scoping review identified 145 different interventions, which we described and catalogued in an interactive online database https://eppi.ioe.ac.uk/eppi-vis/Review/Index/762 [8]. These interventions primarily targeted change in different factors influencing parent carer behaviour (e.g., self‐efficacy, self‐determination, self‐management, advocacy, health literacy and wellbeing).

Despite legislative commitment and the abundance of initiatives aimed at supporting different aspects of parent carer empowerment, parent carers continue to report feeling disempowered when trying to advocate for their child [16, 17, 18]. In order to better understand this enduring problem, we conducted a qualitative study to explore the contextual factors that influence parent carer empowerment from the perspectives of parent carers, children's healthcare service providers, managers and commissioners [19]. We identified and described a complex system of interrelated factors that seem to influence both parent carers and service providers, shaping how these roles are experienced and performed, and ultimately determining whether parent carers are empowered or disempowered. Importantly, this highlighted that empowerment interventions directed solely at parent carers are unlikely to lead to long‐term sustainable change. Instead, we suggested that change needs to happen in other areas of the complex system that currently disempowers them.

There is a significant gap around initiatives or interventions that target change mechanisms across different parts of the complex system that influence parent carer empowerment (e.g., changes at an organisational/institutional level or changes to service provider behaviours and actions). This gap informed the focus of this study, which uses a participatory, theory‐driven method called Theory of Change (ToC) to identify and prioritise areas in the complex system that need to change to empower parent carers.

Theory of change (ToC) is ‘an outcomes‐based approach which applies critical thinking to the design, implementation and evaluation of initiatives and programmes intended to support change in their contexts’ [20]. It uses a backward‐mapping process that starts with the intended impact or long‐term outcome, and then sets out a causal pathway describing the necessary change processes, and the short‐term and medium‐term outcomes required to achieve it [21]. Importantly, a ToC articulates a theory of how, why and for whom an intervention is expected to work, which can be tested by assessing progress against outcomes at each stage of the causal pathway [22].

Incorporating ToC into health‐focused intervention design aligns with the Medical Research Council (MRC) framework for developing and evaluating complex Interventions [23]. De Silva and colleagues suggest that ToC may strengthen the MRC guidance by providing a methodological structure for deeper participatory engagement and by supporting the design of interventions that are explicitly embedded in the local context [22]. Using a ToC approach also ensures that the design and development of the intervention is carried out and documented transparently, allowing the underlying change mechanisms to be clearly understood and facilitating replication, adaptation, and implementation across different settings.

**Figure 1:**
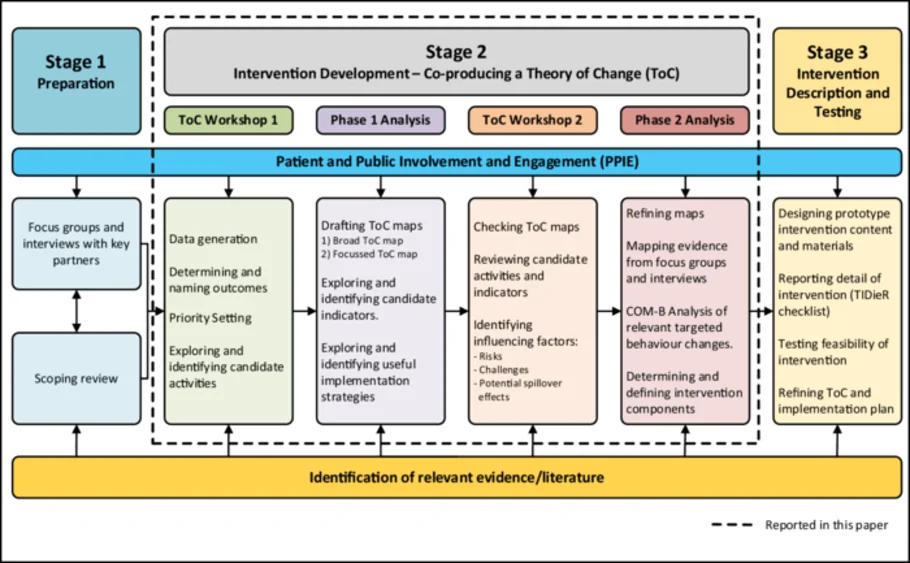
Three stage research programme.

This paper presents Stage 2 of our multi‐stage research programme, which has been designed and delivered in collaboration with a team of parent carer research partners (Figure 1). Building on our scoping review and qualitative study in Stage 1, Stage 2 aimed to co‐produce a ToC to guide the development of an intervention to empower parent carers of children with neurodisability in healthcare settings.

## 2 Methods

Our qualitative study adopted a co‐design approach, incorporating participatory workshops with iterative phases of analysis.

### 2.1 Theoretical Framework and Epistemological Assumptions

Our methodological approach to developing the new intervention is underpinned by both realist evaluation and constructivist theoretical frameworks. It is increasingly recognised that realist and constructivist philosophical positions can be held simultaneously, particularly in applied social research and evaluation [24]. A realist ontology assumes that causal mechanisms and structures exist independently of human perception, and can help explain how and why outcomes occur in specific contexts [25]. On the other hand, a constructivist epistemology acknowledges that knowledge is socially constructed, shaped by individuals' experiences, values, and interpretations [26]. When combined, these positions allow us to explore both the underlying mechanisms driving change (realism) and the subjective meanings and lived experiences of those involved (constructivism).

This critical realist position (ontological realism and epistemological constructivism) has guided our decision to adopt the ToC methodology to develop our intervention. We believe using ToC offers the opportunity to embed participatory approaches to co‐construct change pathways, while maintaining a realist focus on identifying ‘what works, for whom, and under what conditions’ [25]. By integrating both perspectives, we believe our approach has produced the framework for a contextually rich and practically useful complex intervention.

To enhance the deliverability of our intervention, we have incorporated contemporary theoretical approaches from behavioural science into the ToC process, specifically a COM‐B (Capacity, Opportunity, Motivation—Behaviour) behavioural analysis [27]. This approach, which has its roots in the Theoretical Domains Framework (TDF) [28, 29], has allowed us to proactively embed implementation strategies into the intervention design process.

### 2.2 Setting and Sample

Using study eligibility criteria (Supporting Information S1), we recruited 9 participants from a population of parent carers and staff associated with the Children and Families Service of a large NHS organisation in England to become members of our ToC working group. We recruited participants using a range of strategies including; convenience sampling, volunteer response sampling, and purposive sampling. Participants that had been involved in Stage 1 of the project were contacted and offered the opportunity to participate. All potential participants who expressed an interest in taking part were contacted by a member of the research team (J.R.), who gave more detailed information about the study and answered any questions. If they decided to participate, their informed consent was recorded.

Our group comprised a mix of parent carers (*n *= 4), allied health professional (AHP) service providers (*n *= 3), one service manager and one representative from the commissioning service (Table 1).

**Table 1:** Participant demographics.

#	Stakeholder group	Age	Gender	Ethnic background	Highest educational qualification	First language	Child's main diagnosis
1	Parent carer	35–44	F	White, British	GCSE or equivalent	English	Cerebral palsy
2	Parent carer	35–44	F	White, British	Undergraduate degree or equivalent	English	Charcot‐Marie‐Tooth
3	Parent carer	45–54	F	White and Black Caribbean	GCSE or equivalent	English	Cerebral palsy
4	Parent carer	35–44	F	Other Asian background	Undergraduate degree or equivalent	Tamil	Cerebral palsy
5	Service provider	24–34	F	Other White background	Undergraduate degree or equivalent	Croatian	N/A
6	Service provider	45–54	F	White British	Undergraduate degree or equivalent	English	N/A
7	Service provider	35–44	F	White British	Postgraduate degree or equivalent	English	N/A
8	Service manager	55–64	F	White British	Postgraduate degree or equivalent	English	N/A
9	Service commissioner	24–34	F	White British	Postgraduate degree or equivalent	English	N/A

### 2.3 Procedure

The participatory ToC process took place over a period of 4 weeks, consisting of two structured workshops and iterative phases of analysis (Figure 1). The workshops were both co‐facilitated by a researcher (J.R.) and a parent carer partner (S.M.). The analysis was led by the lead researcher (J.R.) in liaison with the research team and parent carer research partners.

### 2.4 ToC Workshop 1

The first ToC workshop was carried out in person over a full day at a hotel conference suite. We ensured that all unsalaried participants were offered remuneration for their time and any travel/childcare costs incurred, as well as regular refreshments and a cooked meal.

Prior to attending, we shared information about the aims and objectives for the workshops and an agenda for Workshop 1 with all participants. During the workshop, we provided brief information about theory of change and empowerment, and a summary of our preceding Stage 1 work (Figure 1). However, most of the workshop involved group participation and interaction, facilitated by structured activities. Throughout the workshop, photographic records and field notes were captured by both workshop facilitators.

#### 2.4.1 Activity 1: Determining and Naming Outcomes

**Figure 2:**
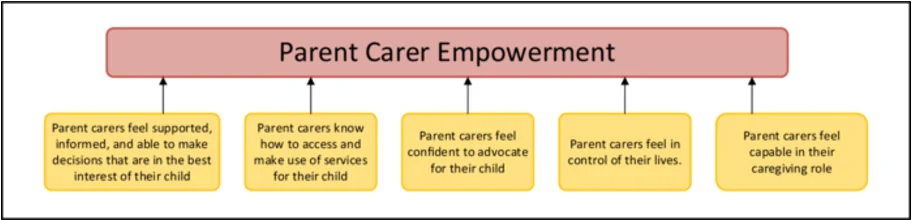
Long‐term outcomes required for parent carer empowerment.

We began Activity 1 by describing our broad vision of parent carer empowerment and by sharing five predetermined long‐term outcomes, based on empowerment literature and the findings from our interview/focus group study in Stage 1 (Figure 2).

**Figure 3:**
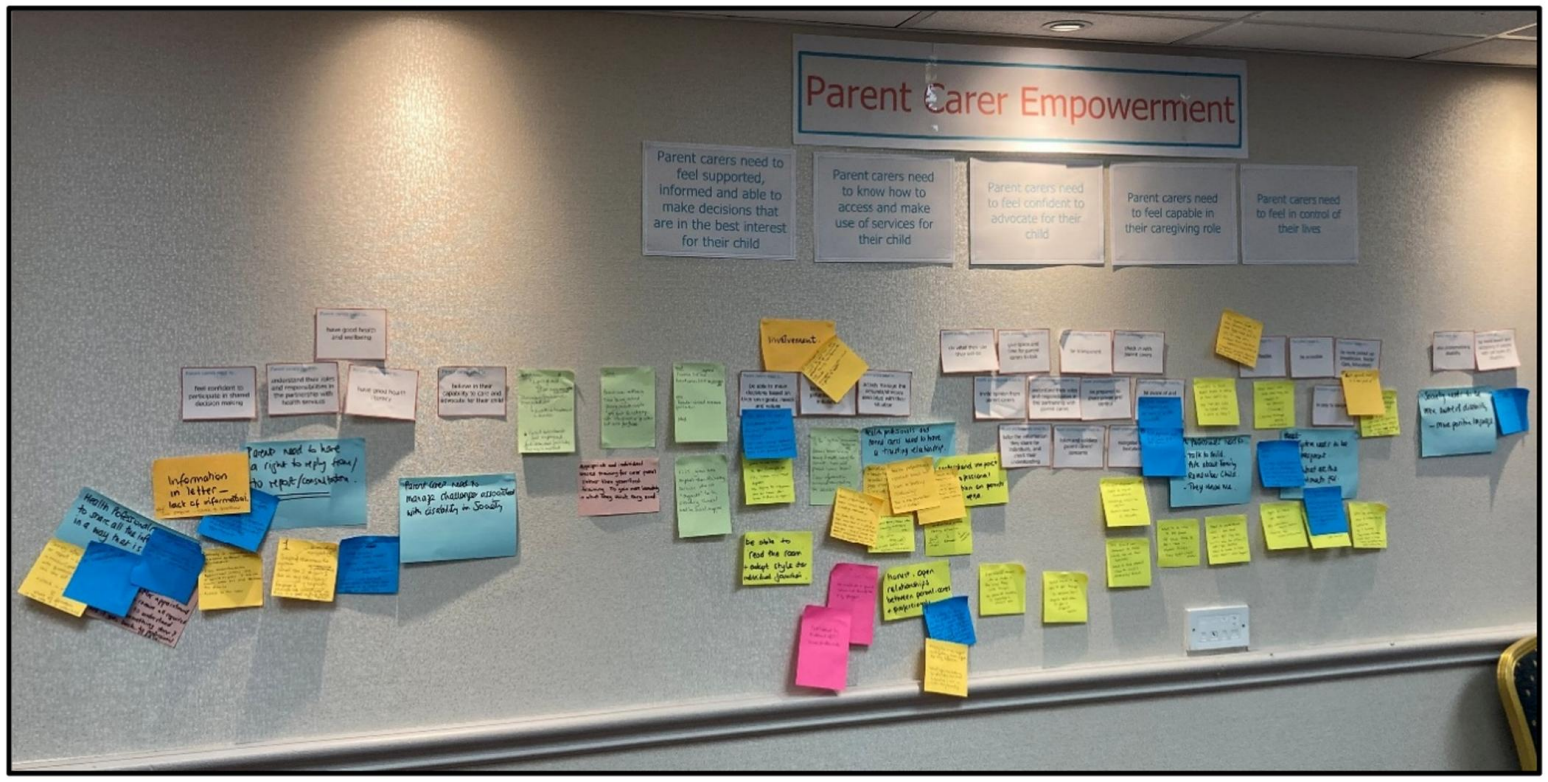
First iteration of the ToC map.

The group then worked collectively, considering ‘what needs to happen?’ to achieve the long‐term outcomes. Discussions were prompted with questions such as ‘who needs to be involved?’ and ‘why does this need to happen?’ All ideas were written on sticky‐notes and stuck to a wall, which became the first iteration of our ToC map (Figure 3). The group was encouraged to read the comments on the other notes, to notice connections, and to begin to aggregate similar ideas by moving the notes around to create tentative causal pathways.

#### 2.4.2 Activity 2: Setting Priorities

The group spent time in discussion, considering which outcomes, groups of outcomes or causal pathways they felt should be prioritised as target areas for change. Each group member cast two votes, including a rationale for their decision, which were recorded by the facilitators.

#### 2.4.3 Activity 3: Exploring and Identifying Candidate Activities

In smaller groups, participants discussed what resources, training, equipment and/or activities might be required to achieve the prioritised outcomes, and to move along the causal pathways. They also discussed external factors and/or conditions that might enable or constrain movement along the causal pathways. The groups created sticky‐note maps and presented their thoughts to the whole workshop.

### 2.5 Phase 1 Analysis

We coded all sticky‐notes and comments (i.e., 1(b)—sticky‐note 1; comment b) and then grouped comments together in themes. Supported by photo records and field notes, we described these groups/codes in terms of short‐term, medium‐term, and long‐term outcomes, which we used to create a broad ToC map. Then we extracted the outcomes/causal pathways prioritised in workshop 1 from the broad ToC map, creating the framework for a focused ToC map. At this point, we also cross‐checked that the prioritised topics aligned with the findings from our Phase 1 qualitative study. For the focused map, we consolidated the outcomes and added further information, including candidate activities/inputs, indicators, and any assumptions made. This information was gathered from ToC group suggestions, our scoping review database, PPIE feedback, and from the broader literature base.

We then used these data to draw together a tentative conceptual plan for a new intervention.

#### 2.5.1 ToC Workshop 2

The second workshop was delivered online. We began by presenting Phase 1 analysis and provisional ToC maps to the group. This allowed an opportunity for checking and challenging the analytic process, and to begin refinement of the ToC maps. We also shared the conceptual plan for the new intervention with the group, prompting discussion about acceptability, appropriateness, feasibility, and likely impact. The group discussed next steps, offering suggestions on suitable preliminary feasibility testing. Detailed notes were taken by the workshop facilitators, which guided the Phase 2 analysis.

### 2.6 Phase 2 Analysis

This phase involved further refinement of the focused ToC map and intervention plan. We began by reviewing all outcomes and causal pathways included in the focused ToC map to identify any specific behaviour changes the new intervention needed to target. We supported this process by mapping data from our Stage 1 qualitative study. Mapping data from Stage 1 participant accounts helped to add richness and nuance to the description of the target behaviours, and to offer greater transparency and rigour to the methodological process.

We then performed a COM‐B analysis of each target behaviour change. This approach helped to systematically identify factors influencing the target behaviours across the domains of Capability (physical and psychological), Opportunity (physical and social), and Motivation (automatic and reflective). Using the COM‐B Behaviour Change Wheel, we identified additional intervention strategies and established behaviour change techniques (BCTs) to be incorporated into the new intervention [30].

These data were then integrated into the focused ToC map, which we used to refine the final iteration of the intervention. We then defined and described the proposed intervention using the Template for Intervention Description and Replication (TIDieR) checklist [31].

### 2.7 Patient and Public Involvement and Engagement (PPIE)

Parent carer partners have been fully involved in the planning and delivery of the study, which we believe has improved methodological integrity and both the quality and feasibility of the ToC maps and intervention plan. Table 2 presents a summary of the group's involvement, described in line with published Guidance for Reporting Involvement of Patients and the Public (GRIPP2‐Short Form) [32].

**Table 2:** PPIE in this study reported using GRIPP2‐SF.

Topic	Summary of involvement
1. Aim	The group of parent carer research partners were encouraged to bring their own lived experience and expertise, with the aim to: Improve relevance, quality and credibility of the study. Plan and deliver the study in a way that optimised involvement of participants from a diverse range of backgrounds. Explore alternative avenues, gain additional insights and offer potential different interpretations in the analysis. Identify ideas for effective dissemination.
2. Methods	Five parent carer partners were involved across all research stages. Group supported by PenCRU family involvement co‐ordinator. Meeting regularly via Zoom Involvement levels negotiated using the Involvement Matrix [33] Contributions included planning delivery strategies, developing materials, co‐facilitating workshops, co‐producing ToC maps, prioritising intervention focus, and shaping intervention design. NB. All group members were provided with necessary IT support, training, and compensation.
3. Study results	Positive outcomes: Improved recruitment and delivery strategies. Co‐produced relevant and accessible workshop plans and materials. Enhanced workshop engagement via co‐facilitation. Guided and informed data analysis, ToC iterations, and intervention development. Contributed to manuscript writing, improving accessibility. Challenges: High time/resource requirements extended study timeline. In‐person workshop limited involvement for those unable to travel.
4. Discussions and conclusions	Given the topic of our research, it was vital for parent carer research partners to have authentic agency, influence and control over the decisions and actions related to this study. As such, we committed substantial planning, time and resources to ensure our PPIE was both robust and meaningful. We also ensured we started engagement early to build relationships and identify training needs.
5. Reflections/critical perspective	Embedded PPIE was a key study strength. Involvement Matrix supported early role negotiation and trust‐building. NHS research passport barrier resolved with Memorandum of Understanding between the hosting NHS trust and the parent carer research partners. Online‐only meetings limited in‐person relationship building but enabled convenience and geographic diversity.

### 2.8 Ethical Considerations

Cambridge South Research Ethics Committee approved the study and research materials (ref‐24/EE/0020). All participants gave their informed consent to take part and were made aware of their right to withdraw at any point without consequence.

## 3 Results

We collected 111 Post‐it notes, containing 133 comments and suggestions. These were sorted into 12 groups, as described above. Within these groups, we identified and described 13 medium‐term outcomes, 29 short‐term outcomes and two additional long‐term outcome (Table 3).

**Table 3:** Identifying and naming outcomes.

Group	Outcomes	Audit trail to notes
(A) A personable approach	Medium‐term: service providers adopt a personable approach towards parent carers	9(a), 9(b), 12, 13(a), 13(b), 13(c), 17, 23(a), 23(b), 23(c), 23(d), 25(a), 25(b), 38(a), 38(b), 38(c), 45, 66.
	Short‐term: service providers know the family	
	Short‐term: service providers understand family's unique circumstances	
	Short‐term: service providers are considerate of family's time	
(B) Recognising limitations	Medium‐term: service providers recognise their limitations	26(a), 26(b), 30(a), 30(b), 51, 59.
	Short‐term: service providers are willing to change after feedback	
	Short‐term: service providers are open and honest about the scope of their role and their limitations	
	Short‐term: service providers seek appropriate support from other agencies	
(C) Addressing the power imbalance	Long‐term: there needs to be a change in professional culture related to the privileging of medical knowledge	6, 7, 18, 19, 54, 55, 56, 68.
	Medium‐term: the power imbalance/dynamic between parent carer and service provider is addressed	
	Short term: service providers need to mitigate for the power dynamic	
	Short‐term: parent carers and service providers need to be aware of, and acknowledge the power dynamic	
(D) Supporting authentic involvement	Medium‐term: service providers need to support authentic involvement of parent carers	3, 11, 26(b), 27, 28, 33, 39(a), 39(b). 39 (c), 46, 49, 50, 52, 61.
	Short‐term: service providers need to tailor programme/expectations to be manageable for families	
	Short‐term: service providers should be flexible/adaptable to family needs	
	Short‐term: service providers need to authentically listen to parents and validate their concerns	
	Short‐term: service providers need to be accessible	
	Short‐term: service providers need to be accountable	
(E) A trusting relationship	Medium‐term: service providers and parent carers have a trusting relationship	48, 53, 58, 60, 62.
(F) Systems	Medium‐term: systems are clear and easy to navigate	1(a), 1(b), 4, 8, 10(a), 10(b), 14, 15, 16, 20, 24(a), 24(b), 31, 67, 75.
	Short‐term: parent carers understand the systems	
	Short‐term: service providers understand the systems	
	Short‐term: systems are flexible	
	Short‐term: systems are joined up (health, social, education)	
	Short‐term: systems are accessible	
(G) Parent carer capacity and capability	Medium‐term: parent carers participate in shared decision‐making based on their own goals, needs and values	2, 34, 35, 36, 37, 57, 71.
	Medium‐term: parent carers believe in their capability and recognise their expertise	
	Short‐term: parent carers have good health and wellbeing	
	Short‐term: parent carers actively manage the demands/stresses associated with their situation	
(H) Culture	Long‐term: societal culture change towards disability	22(a), 22(b), 40, 41.
(I) Accessible health professional	Medium‐term: service providers are readily/easily accessible to parent carers	11, 32(a), 32(b), 21, 66.
	Short‐term: there is regular contact service providers and parent carers	
	Short‐term: service providers respond quickly	
	Short‐term: a known service provider is contactable	
(J) A right to respond	Medium‐term: parent carers have a right to respond to service provider reports/consultations	42, 74, 76, 77, 79, 83, 84.
	Short‐term: there is a system in place for parent carers to be able to respond to reports/consultations and received feedback	
	Short‐term: parent carers have the opportunity to ask questions before, during and after consultations	
	Short‐term: reports/letters are produced quickly	
	Medium‐term: (linked to group J and K). Parent carers have good health literacy	
(K) Sharing information		5, 69, 70, 72, 73, 78, 80, 81, 82, 85, 86, 87, 88, 89.
	Medium‐term: service providers share information that is understandable	
	Short‐term: information is tailored for individuals	
	Short‐term: information is shared prior to consultation	
	Short‐term: information is all in one place and accessible	
	Short‐term: information about content of consultation is shared quickly	
(L) Therapeutic partnership	Medium‐term: both parent carers and service providers need to understand their roles and responsibilities in the therapeutic partnership	47, 65

**Figure 4:**
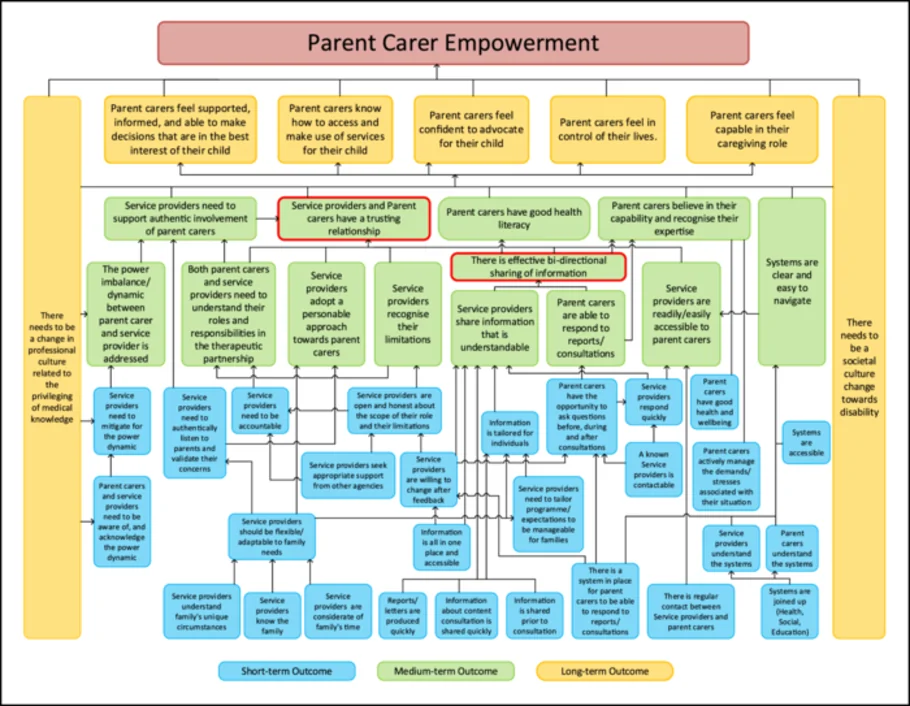
The ‘broad’ ToC framework.

Figure 4 presents all of the short, medium and long‐term outcomes, their connections and causal chains co‐created by the participants and researchers in our ‘broad’ ToC Framework.

Two related medium‐term outcomes were prioritised by the group as the focus for the new intervention (highlighted in Figure 3 with red borders): (1) Effective bi‐directional sharing of information and (2) Health professionals and parent carers have a trusting relationship. Analysis of the causal pathways associated with these outcomes identified seven target behaviour changes for service providers to be incorporated into the intervention. Table 4 presents the seven target behaviours along with the linked intervention types/strategies and behaviour change techniques (BCTs) identified in the COM‐B analysis. A full record of the COM‐B analysis, including data mapped from the Stage 1 qualitative study, is provided in Supporting Information S2.

**Table 4:** The seven target behaviour changes.

Identified target behaviour change	Linked intervention types/strategies	Linked behaviour change techniques (BCTs)
Service providers need to acknowledge and mitigate the power imbalance	Education Training Enablement Persuasion Incentivisation Coercion Environmental restructuring Modelling Restriction	1. Credible source 2. Framing/reframing 3. Salience of consequences 4. Prompts and cues 5. Restructure physical environment 6. Feedback on outcome(s) of behaviour 7. Behavioural experiments 8. Incompatible beliefs 9. Instruction on how to perform a behaviour. 10. Information about consequences 11. Problem solving 12. Feedback on behaviour 13. Habit formation 14. Self‐monitoring behaviour 15. Information about social and environmental consequences 16. Information about health consequences
Service providers need to be flexible and adaptable, tailoring support to families' individual needs		
Service providers need to share information that is understandable		
Service providers need to be willing to change following feedback		
Service providers need to be open and honest about the scope of their role and their limitations		
Service providers need to authentically listen to parent carers and validate their concerns		
Service providers need to adopt a personable approach towards parent carers		

**Figure 5:**
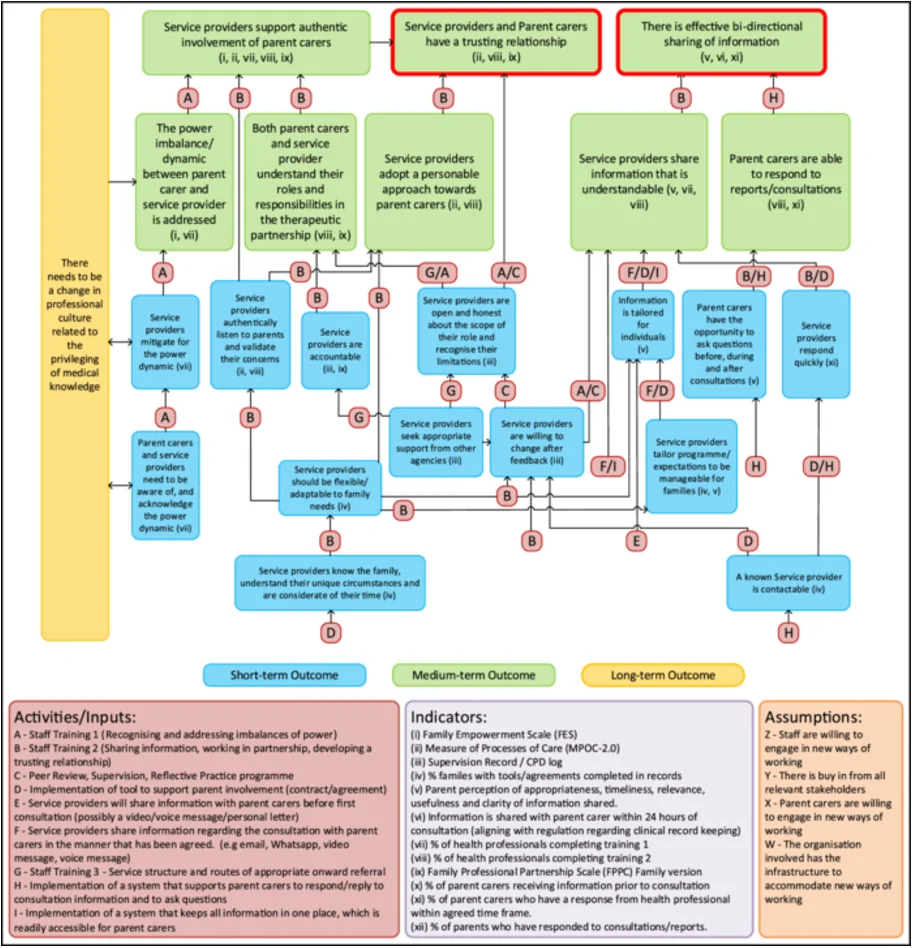
The focused ToC map.

The final iteration of the focused ToC map is presented in Figure 5.

The proposed new intervention framework focuses on influencing change in service provider behaviours, actions and interactions by incorporating the BCTs identified in the COM‐B analysis. The intervention is comprised of three main components: (a) Two service provider workshops, (b) three practice changes, and (c) structured supervision. A summary of the intervention structure and content is presented in Table 5.

**Table 5:** Summary of the proposed intervention.

Intervention component	Description
a. Two service provider workshops	Workshop 1—Working with parent carers: Recognising and addressing imbalances of power. Key learning objectives: To understand what is meant by an imbalance of power, including noticing how it is created and maintained, in relation to working with parent carers. To explore additional factors that influence the power imbalance. To understand the benefits of addressing the power imbalance. To identify some strategies to address the power imbalance within a clinical relationship. Workshop 2—Working with parent carers: Working in partnership, sharing information and developing a trusting relationship. Key learning objectives: To review the concept and theoretical underpinnings of family‐centred practice. To understand the challenges with offering personalised care. To recognise the value of personable care. To introduce a ‘new way of working’, consisting of three changes to current practice, that have been designed to help empower parent carers. Both workshops will be delivered live, either in person or online. They will consist of pre‐recorded content (including video testimony from parent carers and case study examples), provision of information and guided discussion.
Linked BCTs: (Table 4) 1, 2, 3, 7, 8, 10, 11, 14, 15, 16.	
b. Three practice changes	1. Partnership agreement: A document written collaboratively by the health professional and parent carer, during the child's first appointment. This will set out the terms and expectations of the therapeutic partnership. 2. **I**ndividualised message before first consultation: Prior to a child's first appointment, health professionals will send a photo and a voice message introducing themselves and their role to the parent carer. 3. Sharing a summary of information after the appointment: This would include brief bullet points and key takeaway messages from the consultation, shared in the way requested by the parent carers, within an agreed time (ideally within 24 h).
Linked BCTs: 4, 5, 9, 13	
c. Structured supervision	This will be linked to existing supervision processes within the organisation. There will be time allocated to discuss how changes to practice are being managed, specifically how staff are responding to feedback and adapting to families' individual needs.
Linked BCTs: 6, 12, 14	

A more detailed provisional description of the proposed intervention is provided using the TIDieR checklist (Supporting Information S3).

## 4 Discussion

This study presents the framework for our new intervention, which aims to empower parent carers, primarily by targeting change in the behaviours, actions and interactions of service providers. The intervention is underpinned by a rigorously co‐produced Theory of Change, which was developed in partnership with a group of parent carers, service providers, managers and commissioners, parent carer research partners and researchers. As such, we believe our intervention is likely to deliver sustainable change in a manner that is acceptable, appropriate and feasible to those it involves in practice. In this section we will discuss the intervention in the context of existing literature and describe implications for future work in light of the strengths and limitations of the study processes.

### 4.1 The Intervention

Many interventions have been developed to address various aspects of parent carer empowerment, but most target changing parent carer behaviours. Details of these programmes can be found in our scoping review and interactive online database, which was developed in Stage 1 of the research programme [8]. Our new intervention takes an alternative approach by targeting barriers to parent carer empowerment that arise specifically from service providers' behaviours, actions and interactions with parent carers.

The intervention content and target behaviour changes have been shaped by the ToC process and the resulting ToC maps and are supported by data mapped from the qualitative study. The structure and delivery strategies for the intervention have been informed by our COM‐B analysis, which guided selection of the BCTs that have been incorporated across the three complementary components.

#### 4.1.1 Component 1: Two Service Provider Workshops

A key element of the facilitated workshops is to raise service providers' critical consciousness of the power structures and factors that might influence how they behave, act and interact with parent carers. Drawing on principles of critical pedagogy, it employs a problem‐solving, action‐oriented approach to problematise these power structures and to create space and opportunity for change [34, 35, 36]. This approach will require service providers to critically reflect on the impact and influence of their own values, beliefs, behaviours and interactions, as well as the influence of the wider organisational, institutional and societal context [37].

We acknowledge that our commitment to fostering this type of critical reflection as a mechanism of change is not without risks. Previous studies have found that when reflection is mandated, it can be met with resistance from healthcare staff [38, 39], and may become superficial, guarded, or simply used to rationalise existing practices [37]. Furthermore, authentic critical reflection can create significant discomfort for service providers; not only because of potential negative perceptions associated with being self ‘critical’ [37], but also because critical thinking intentionally disrupts the status quo and demands service providers to question their existing, established practices [35]. In the context of our intervention, critical reflection may even reveal service providers' complicity in the power structures that disempower parent carers [40].

For these reasons, the workshops will need to be designed and delivered judiciously, by trained facilitators who have robust knowledge of the theory underpinning critical reflection. In particular, this will involve using critical theory to focus attention on how roles and identities of service providers are constructed, rather than on the practices of individual service providers [35].

#### 4.1.2 Component 2: Three Practice Changes

To close the loop of reflection, to stop it being merely rumination, action is required [36, 38]. Freire refers to this process of reflection and action as praxis [34]. The second component of the intervention is designed to scaffold this process by giving service providers a potential mechanism to operationalise their critical reflection and learning. We recognise that it would be favourable (and likely more effective) for service providers to develop their own solutions to move their reflection and learnings into practice. However, our COM‐B analysis informed a pragmatic decision to include the three ‘proposed’ practice changes, as a strategy to actively drive the desired behaviour change. Our expectation is that service providers will adopt the proposed practice changes flexibly, adapting and applying them in ways that fit their own context.

#### 4.1.3 Component 3: Structured Supervision

The third component of our intervention is structured supervision. Clinical supervision has been found to have direct benefit on processes of care [41], and on person‐centredness [42]. It is increasingly being used as a tool to support the implementation, adoption and sustainability of new evidence‐based interventions [43]. The inclusion of structured supervision is further supported by some of the BCTs identified in our COM‐B analysis, specifically: (6) [giving] feedback on outcome(s) of behaviour; (12) [giving] feedback on behaviour; (14) self‐monitoring of behaviour.

### 4.2 Implications for Practice and Future Work

Our study has produced both a ToC for parent carer empowerment in healthcare settings, and the framework for a new multifaceted intervention to deliver change in the areas of the ToC prioritised by our participant group.

We believe these outputs offer a substantive contribution to the parent carer empowerment literature and provide a valuable resource for service providers, managers, and commissioners seeking to explore parent carer empowerment within their own organisations. Whilst our ToC and the intervention were developed for a specific healthcare context, our transparent processes, strategies, and reporting mean that both can be readily replicated and/or adapted for use in other settings. This may involve prioritisation of other areas of the broad ToC map not addressed in our intervention, which could inform the development of alternative new initiatives to target other factors influencing parent carer empowerment.

Stage 3 of our research programme (Figure 1) will include preliminary proof‐of‐concept testing of the proposed intervention, which will guide ongoing refinement of both the ToC and the intervention itself. This early testing will involve further engagement with parent carers and professionals and use an appropriate implementation science framework to identify potential barriers and levers for implementation. This process will support continued development of a robust implementation plan to facilitate effective delivery of the intervention in our real‐world clinical setting.

### 4.3 Strengths and Limitations

A key strength of our research, and the resultant intervention, is the robust development and transparent reporting of the ToC, both in terms of process and outputs. Combined with our commitment to PPIE, we believe this has led to the co‐production of a new intervention that is grounded in theory, evidence and lived experience. We also offer a clear rationale for each component of the intervention and a detailed plan for ongoing iterative development, optimisation and evaluation. This is strengthened further by the integration of a COM‐B analysis within the ToC and intervention development processes. This contemporary approach has helped to embed a provisional implementation strategy into the design of the intervention, increasing the likelihood that it will be acceptable, deliverable and effect change in the target behaviours.

Another strength of the study was the impact the participatory workshops had on the parent carers who took part. Feedback given to the parent carer workshop facilitator (SM) was that the workshops offered a space where the participants felt listened to, respected and empowered.

A potential limitation of our study is that the proposed intervention is specific to the context in which it has been developed, meaning it will need to be carefully adapted for use in other settings. Nevertheless, the transparent design process and publication of the ToC maps will support any adaptation/replication efforts for other contexts.

Another limitation is that participant numbers in our ToC workshops were small and there was only one group. Furthermore, three of the four parent carer participants were parents of children with cerebral palsy. As such, the outputs of the group may not be representative of the broader cohort of parent carers of children with neurodisability. Additional workshops, with different participants, may have generated a wider variety of views and opinions and provided further opportunities to critique the ToC maps produced. We feel this limitation was mitigated somewhat by ongoing consultation with the expert research team and broader PPIE group before, during and after the workshops were conducted.

## 5 Conclusions

The development of a Theory of Change to empower parent carers of children with neurodisability in a healthcare setting has informed the design of a novel, co‐produced, theory‐driven and evidence‐based intervention. The intervention targets change in service provider behaviour by focusing on self‐awareness and critical reflection, specifically related to how information is shared/managed and how interactions with parent carers are framed and performed.

## Author Contributions

**Jim Reeder:** conceptualisation, investigation, funding acquisition, writing – original draft, writing – review and editing, data curation, formal analysis, project administration, methodology, resources. **Saira Minhas:** conceptualisation, investigation, writing – review and editing, resources, formal analysis, data curation, validation. **Erica Breuer:** conceptualisation, methodology, writing – review and editing, supervision, validation. **Jane R. Smith:** conceptualisation, writing – review and editing, supervision, methodology, validation. **Sally Kendall:** conceptualisation, methodology, validation, writing – review and editing, supervision. **Christopher Morris:** conceptualisation, methodology, validation, writing – review and editing, writing – original draft, supervision.

## Ethics Statement

Cambridge South Research Ethics Committee approved the study and research materials on 08/02/24 (ref24/EE/0020).

## Conflicts of Interest

The authors declare no conflicts of interest.

## Supporting information


Supporting File 1

Supporting File 2

Supporting File 3

## Data Availability

The data that support the findings of this study are available on request from the corresponding author.
