# Supplementary material for: Empowering Parent Carers of Children With Neurodisability in Healthcare Settings: Co‐Producing a Theory of Change for Intervention Development
Source: Health Expect. 2026 Sep 9;29(5):e70871. doi: 10.1111/hex.70871 (PMC13556309; doi:10.1111/hex.70871)
Supplement: Supplementary file 1 [file HEX-29-e70871-s002.pdf]

#	Behaviour	Who it involves	Influencing factors			Additional Evidence:	Linked Interventions:	Potential Linked BCTs:	Intervention component:
	(taken from ToC)		(COM-B category)		(Description)	(taken from qual data)	(types table)	(mapping table)	(Operationalisation of BCTs)
1	Acknowledge and mitigate for power imbalance	Service Providers	Capabilities	Physical					Education/Training: Content should include: 1) Information about how the power imbalance is created and maintained, and exploring what alternative perspectives might offer (this needs to include an acknowledgment that equity in the therapeutic relationship may be impossible and perhaps undesirable); 2) Information about the benefits (consequences) of mitigating for the power imbalance, both for parent carers and for service providers (this needs to include potential health benefits, social benefits and environmental benefits); 3) Information on how to identify factors that might influence mitigation of the power imbalance, and to select strategies that increase facilitatory factors and reduce barriers; 4) Prompting detailed planning of **HOW** to mitigate for the power imbalance (Implementation Intentions) - this would include addressing concerns related to added work load; restructuring the physical environment to minimise influence on 'power'. *Strategies to enhance impact of training* should include: 1) Using stories and testimony from parent carers (as credible sources of information); 2) Highlighting the incompatibility between desire to empower parent carers and the persistence of 'professional as expert' discourse; 3) Emphasis on the beneficial consequences (need to go beyond just giving the information); 4) Use of posters in the clinic room to remind health professionals to practice this behaviour. Monitoring/Maintaining behaviour: Encouragement of structured supervision and reflection on performance of 'power-mitigating' behaviours and actions; soliciting feedback from parent carers.
				Psychological	1) There is a need to understand about the power imbalance, the factors that cause/create it and other factors which might influence it. 2) There is a need to understand what changes in practice might mitigate for the power imbalance. 3) There is a need to understand what it might look like when the power imbalance has been addressed.	"…So, for example, if I go to a doctor I always consider him as a bit superior to me. He's the expert and he's going to tell me things, and I'm going to do what he says" - OSH8 "There are certain circumstances where, no, they wouldn't, they would probably still be feeling like, 'Well actually, I'm the expert,' and I suppose maybe intimidated by letting go of their expertise, because that's what defines you." - OSH2	Education, Training, Enablement	1) Information about health consequences; 2) Information about social and environmental consequences; 3) Information about emotional consequences; 4) Problem solving; 5) Action planning; 6) Self monitoring of behaviour.	
			Motivation	Automatic	1) The default behaviour is for service providers to take on the position of decision maker and fixer and parent carer taking on position of passive recipient of care. This is the line of least resistance and is generally left unchallenged. (linked to culture). 2) The status quo seems to be underpinned by a strong motives/instincts/habits from service providers to 'help', to provide care and to fix (from a point of view of an expert). This itself is driven (in part) by parent carers desire/instinct/habit to 'be helped'. It may be the 'change' should try to move service provider motives from 'helping' to 'supporting' parent carers to help themselves and their child.	"Actually I think many parents are the expert on their children and I think we – I'll use the royal we for health professionals – can sometimes feel like we know more than them." - OSH10. "cause sometimes I have had people go, well I went to the doctors and they said…well what do you think it is and I said, well, you're the doctor so you should tell me." - OSH13 "…yeah I think for parents to be ready and able to be able to take up that role is a bit more of a shift for some parent carers than it is for others…and it probably depends on so many different factors…whether or not you feel like that's…that is your place to do it, whether you feel capable and able to do it…I think for some parent carers it is a…I just don't know and I just want you to help…you know more…please…" - OSH5 "You accept it because they have a title that is supposed to put them in a level of authority over your child's health I suppose, an authority of health. I don't know if that's the right term but you accept what they're saying because you think they know. But what I've really learned is, it's not that they don't know, they're just not always right." - P7	Persuasion, Incentivisation, Coercion, Training, Environmental restructuring, Modelling, Enablement.	1) Credible source; 2) Framing/Reframing; 3) Salience of consequences; 4) Prompts and Cues; 5) Restructure physical environment.	
				Reflective	1) There appears to be lots of good intention to address the power imbalance; however this sits in tension with deep seated discourses related to 'professional as expert' and perceived restrictions related to the structure of services (e.g. clinical pathways, standardised assessments, etc).	"Because some feel that, I think some of it could be cultural, but I think some carers, some parents, some carers, feel that they don't know and they need to be told what to do but, actually, I think they're just not, culturally or historically not used to that, some of them." - OSH12	Education, Persuasion, Incentivisation, Coercion, Modelling.	1) Feedback on outcome(s) of behaviour; 2) Behavioural experiments; 3) Incompatible beliefs	
			Opportunities	Social	1) There are powerful social norms/discourses related to privileging of medical training and knowledge. These are situated in both professional culture and societal culture. 2) The status quo, which is professionals making decisions and parent carers being passive recipients of care, seems to be the expectation of both groups. 3) Professional status is still regarded highly.	"…I think probably the medical training is better than it used to be, but there's still quite strong hierarchies within medicine and although we've moved away hopefully from a lot of paternalistic medicine, there is still this kind of power imbalance sometimes between the doctor and parents." - OSH13	Restriction, Environmental Restructuring, Modelling, Enablement.	1) Framing/reframing	
				Physical	1) There is a sense that working in a new way would require more time and resources for service providers, who are already under significant strain.	"…I think the time limits on some appointments are ridiculous. I think the way the clinics – I'm just thinking of my work but having a very set time that you can only see somebody within and then it's straight onto the next person, that doesn't allow for any thinking time…" – OSH10	Training, Restriction, Environmental Restructuring, Enablement.	1) Instruction on how to perform a behaviour.	
2	Be flexible and adaptable, tailoring support to families' individual needs	Service Providers	Capabilities	Physical					Education/Training: Content should include: 1) Presentation of the complex system of factors and actors (context) that influence the parent carer experience - suggesting the potential benefits of using this model as a lens to explore the parent carer's individual circumstances (and the potential intended and unintended consequences of disruption to the complex system - from an intervention/behaviour/etc); 2) Brief reminder about the value of family centred practices; 2) Clear strategies how to operationalise family centred practices - specifically how to acknowledge and adapt to family's unique circumstances; 3) Highlighting the barriers and facilitators for family centred practices (why has it worked/not worked in the past) and workshoping solutions; 4) Introduction of possible new habits (contacting before consultation), sharing information in a way that is preferred by families (acknowledging this is 'more' work), giving families a clear mechanism to respond to consultations and to ask questions. *Strategies to enhance impact of training* should include: 1) Using stories and testimony from parent carers (as credible sources of information); 2) Offering clear instruction how to do this behaviour and how to monitor performance; 3) Signposting to resources (e.g. F-words hub); 4) Use of posters in clinic rooms to remind health professionals to practice this behaviour. Practice Changes: 1) Introduce 'Brief Agreement' regarding information sharing. This needs to be short, concise and easy to complete. 2) Introduce sharing of information prior to consultation (what to expect, who they will see, questions to think about, etc). This needs to be offered in a personal way e.g. video message; 3) Introduce sharing of information following consultation with encouragement to parent carer to respond/ask questions. Monitoring/Maintaining behaviour: Encouragement of structured supervision and reflection on performance of 'adaptive family centred' behaviours and actions; soliciting feedback from parent carers.
				Psychological	The concepts related to family centred practice are well known and generally well accepted. Service providers need to know how to operationalise these concepts.	"…so the NHS definitely have a big push on personalised care, so the theory is there but, operationally, it's extraordinarily challenging…" - OSH9 "…They put barriers in the way, what's the research behind it? What's the evidence? Has it been through NICE guidance? Is it MRHA, or whatever?…" – OSH9	Education, Training, Enablement	1) Instruction on how to perform the behaviour; 2) Information about consequences; 3) Problem solving	
			Motivation	Automatic	It seems to be habitual/instinctive for service providers to allow themselves to be constrained by the 'systems' within which they operate (computer systems, clinical pathways, etc). This can lead to more generalised (rather than individualised) support. It may be that a change required would be a movement towards a prioritisation of individualised care over adherence to systemic constraints.	"But isn't it about resources ultimately because I do feel our services have become more centralised and squeezed. Previously where I used to work you could have gone out to home visits. It was pretty much as long as the family needed it but there's very set pathways I think now; six sessions and that's your limit." - OSH10	Persuasion, Incentivisation, Coercion, Training, Environmental restructuring, Modelling, Enablement.	1) Feedback on behaviour; 2) Credible source; 3) Prompts and cues; 4) Habit formation; 5) Restructuring the physical environment.	
				Reflective	There is good intention from service providers to work in family centred ways; however this competes with other motivations (to adhere to recognised/expected standards; to deliver 'best' care; to work within the systems; etc…).	"…I think that is a really sticky point for me because… when a child comes to you and the parent says [what they want to achieve]…OK great that is patient centred and I will obviously make sure that I try to do that…but realistically I am going to do the same thing I was going to do anyway, because I know and have that knowledge…so that feels wrong in my head…because I know we are trying to be really patient centred…" – OSH3	Education, Persuasion, Incentivisation, Coercion, Modelling.	1) Behavioural experiments; 2) Feedback on behaviour.	
			Opportunities	Social	There seems to be strong commitment from stakeholders to offer flexible/individualised support. This is from all levels - institutional/organisational/individual. FCP is seen as a 'good' thing by all.				
				Physical	There are some systems that seem to constrain health professional's ability to work in more individualised ways. The financial pressures on the NHS demand more granular, clarity about how resources/funds are used - this can be very difficult if always offering bespoke care. It is easier to 'price up' packages of care/care pathways - which can be more generic.	"there's very clear time limits on appointments and some professionals like half an hour. We maintain in ours the minimum and we have to stick to that but we can always get people back. I do think time is a huge one" - OSH10 "we are at such deficits that, in many ways, we're behaving, or beginning, having to behave like private industry where you're having to account for every penny 'cause we've gotta make a saving, gotta make - private industry call it profit, we are just trying to get up to breaking even." - OSH9	Training, Restriction, Environmental Restructuring, Enablement.	1) Prompts and cues; 2) Instruction on how to perform the behaviour.	
3	Share information that is understandable	Service Providers	Capabilities	Physical					Education/Training: Content should include: 1) Information about current methods of information provision and how this is controlled by health professionals; 2) Information about the consequences of not sharing information in a way that is understandable; 3) Suggestions for different methods/approached for sharing information; 4) Problem solving issues related to 'time burden' and recording different way of sharing information (e.g. whatsapp, email, SMS, video note, voice note, etc); 5) How to solicit feedback from parent carers about information shared. *Strategies to enhance impact of training* should include: 1) Using stories and testimony from parent carers (as credible sources of information); 2) Use of posters in the clinic room to remind health professionals of desired behaviour. Practice Changes: 1) Introduce 'Brief Agreement' regarding information sharing. This needs to be short, concise and easy to complete. 2) Introduce sharing of information prior to consultation (what to expect, who they will see, quetsions to think about, etc). This needs to be offered in a personal way e.g. video message; 3) Introduce sharing of information following consultation with encouragement to parent carer to respond/ask questions. Monitoring/Maintaining behaviour: Encouragement of structured supervision and reflection on performance of 'information sharing' behaviours and actions; soliciting feedback from parent carers.
				Psychological	Service providers need to know more about the parent carers information needs - which method shared to share, when, what detail, what level of literacy, etc... They also need to understand that information shared in a consultation can be difficult to take on for parents managing their child.		Education, Training, Enablement	1) Instruction on how to perform the behaviour; 2) Information about consequences; 3) Problem solving	
			Motivation	Automatic	1) Service providers habitually record information in clinical records and share a written report with parent carers. This is underpinned by historic professional and organisational standards. 2) There are some pockets of practice where information is shared in other ways; however the report is still seen as the gold standard. 3) It is also habitual for service providers to control the content and flow of information.	"...I don't know. I don't know if it's — I always ask and I always say, 'Oh can you pop that in an email to me? Can you do this, can you do that?' I don't know, it seems like there needs to be, like the NHS App, but one for parents, that access for their children." - P12	Persuasion, Incentivisation, Coercion, Training, Environmental restructuring, Modelling, Enablement.	1) Habit formation; 2) Framing/ Reframing	
				Reflective	Service providers generally believe that they do produce information for families that is understandable; however families have reported mixed feelings about the helpfulness of the information shared.	"...So I tend to think that that is their information, so I share it. What I do do is I suppose there's a – there is a screening process of kind of what I think's gonna be useful and I suppose there is an element of the hierarchy to have me deciding what's useful for parents...." OSH13 ...I have definitely experienced lots of people where they just want to know...tell me information...but actually its...sometimes it isn't having more information, it is about understanding the information they already have... - OSH5	Education, Persuasion, Incentivisation, Coercion, Modelling.	1) Feedback on Behaviour; 2) Behavioural experiements; 3) Credible source.	
			Opportunities	Social	There is good intention from service providers to share information that is understandable (and accessible). This is also encouraged by professional and organisational stakeholders.				
				Physical	Service providers need a functional system that facilitates timely sharing of information with parent carers. This needs to work well alongside other exixting systems to avoid duplication/additional administrative load. 2) Service providers also need to offer opportunity for parent carers to respond and ask questions about ANY information shared - this requires clear availability and timely sharing of information.	"...No, and like I say, even if there was a portal or something that we can access when we want to, you know, I might have an appointment today and look at it in three weeks' time and think, oh, what did they say?" - P12	Training, Restriction, Environmental Restructuring, Enablement.	1) Problem solving; 2) Instuction on how to perform the behaviour; 3)	
4	Be willing to change following feedback	Service Providers	Capabilities	Physical					Education and training: Content should include: Reminder of clinical reflection strategies with emphasis on critical self-reflection and action planning/change. Practice Changes: Introduce mechanism for parent carers to respond to information shared with encouragement to give feedback on service offered and performance of 'wanted' service provider behaviours. Monitoring/Maintaining behaviour: Encouragement of structured supervision and reflection on performance of 'acknowledging limitations and being open and honest' behaviours and actions; soliciting feedback from parent carers.
				Psychological	Reflection and experiential learning are integral to contemporary professional training.				
			Motivation	Automatic	It can be instinctive for service providers to take on the role of fixer/knower/holder of answers - see earlier entries. This can lead to feedback being misinterpreted as criticism or challenge. This needs to be reframed.		Persuasion, Incentivisation, Coercion, Training, Environmental restructuring, Modelling, Enablement.	1) Prompts and cues; 2) Framing/reframing; 3) self-monitoring of behaviour	
				Reflective	Service providers want to be competent (and to be seen as competent) and it can take time/experience for them to recognise this also involves recognising limitations (see below).	"Then this other consultant saw him in emergency situation and it was just her attitude and the way she would listen and the way she would say 'what do you think should happen?' She was very open for saying she wasn't the expert" - P7 "...Not having doubt in front of them, looking confident, because at the end you come together and then you have a nice conclusion to give them, don't you?" - OSH12	Education, Persuasion, Incentivisation, Coercion, Modelling.	1) Framing/reframing; 2) Feedback on behaviour; 3) Information about social and environmental consequences.	
			Opportunities	Social	There may be some perceived pressures on service providers to perform to a certain standard and that any feedback requiring action/change may be seen as a faliure to meet that standard.		Restiction, Environmental Restructuring, Modelling, Enablement.	1) Framing/reframing; 2) Problem Solving;	
				Physical	1) Staff already have structure supervision time and also protected CPD time , where they are encouraged to reflect on their practice (and can be uncomfortable for parents). 2) There are some menchanisms in place for parent carers to offer feedback; however this is not seen as a routine process. More typically feedback would be given if there is some discontent. 3) There are no mechanisms for parent carers to offer routine feedback about information shared and/or to ask questions in a structured way.	"...even on the phone to the speech therapist, when she said we are going to do this and this...I...it instantly made me feel sick, because I thought I'm going to have to tell her that I don't...that I am not happy with that...and it waited for the next phone call for me to say actually the last phone call we had, I'm not...I don't think that is going to work..." - P8	Training, Restriction, Environmental Restructuring, Enablement.	1) Feedback on Behaviour	
5	Be open and honest about the scope of their role and their limitations	Service Providers	Capabilities	Physical					Education/Training: Content should include: 1) Practical information about service structures and routes of appropriate onward referrals; 2) Spotlight on professional culture - reframing expertise and competence to include acknowledgment of limitations; 3) Information on the social consequence of not acknowledging limitations (impact on trusting relationship, impact on dependency). Monitoring/Maintaining behaviour: Encouragement of structured supervision and reflection on performance of 'acknowledging limitations and being open and honest' behaviours and actions; soliciting feedback from parent carers.
				Psychological	1) Service providers need to know clearly about the scope of their role and also the structure of the service/systems that they are working within. 2) Service providers need to know about other services (within the trust and elsewhere) that may be helpful for parent carers and families. They also need to knwo how to access/refer into these services.	"so you open up a whole Pandora's box and then, of course, you get the parent that says, 'Actually, we're not managing diabetes because our housing is awful, the mould's on the –,' and, of course, we're doing it as a, we still do it, and we're improving all the time, but not as a multi-disciplinary, multiagency approach..." - OSH9	Education, Training, Enablement	1) Instruction on how to perform the behaviour.	
			Motivation	Automatic	It is not habitual to question/challenge and self reflect on practice. It seems to be more intinctive to want to maintain the appearance of knowledge/skill/understanding.		Persuasion, Incentivisation, Coercion, Training, Environmental restructuring, Modelling, Enablement.	1) Prompts and cues; 2) Framing/reframing;	
				Reflective	Service providers want to be competent (and to be seen as compent) - possibly underpinned by a mixture of dominant 'professional' discourses, historic expectations and limitations seen as flaws/incompetance/weakness. This seems to outweigh desires to be open and honest about these limitations.	"...I think I would tell myself that it is okay to say 'that's not an approach I'm familiar with. I'm happy to go away and look at that and then shall we pick that up in our next conversation?' I think that would be a key thing rather than worrying that there and then at that point you've got to say something one way or the other. Maybe I might have worried that it was a vulnerability to say I've never heard of this but actually it's just being honest isn't it..." OSH4 " You get far too many people in the medical industry who just think that because they've got a title it sadly makes them think that they are the oracle with all the answers." - P9	Education, Persuasion, Incentivisation, Coercion, Modelling.	1) Framing/reframing; 2) Feedback on behaviour; 3) Information about social and environmental consequences.	
			Opportunities	Social	Whilst recognising limitations is part of the rhetoric of 'good' practice; there is still a sense that competence/knowledge/skill/etc is privileged as 'best practice'. Rather than best practice being linked to self appraisal/reflection/openess and readiness to learn. This is despite there being structured time for supervsion - it may be the way that supervision is structured to support development of the former attributes, as opposed to the latter.	"cause you cannot know it all, as a healthcare professional, across a whole system with very different cultures in how they approach families, as well, different attitudes and understanding of what family's roles are within all of this" - OSH9	Restiction, Environmental Restructuring, Modelling, Enablement.	1) Framing/reframing; 2) Problem Solving;	
				Physical					
6	Authentically listen to parent carers and validate their concerns	Service Providers	Capabilities	Physical					NB This behaviour has considerable overlap/similarities with *'Be flexible and adaptable, tailoring support to families' individual needs'* (see above entry); however there are some additional components to consider. Education/Training: Content should include: 1) Information about the social and environmental consequences of not authentically listening to, and validating, parent carer concerns - including the risk of tokenism/lip service behaviours; 2) Suggested strategies to acknowledge competing commitments and constrainting factors which might impair authentic listening and validating behaviours.
				Psychological	The concepts related to family centred practice are well known and generally well accepted. Service providers need to know how to operationalise these concepts. In particular service providers need to know how to deliver actions related to any raised concerns - input and involvement from parent careres needs to change approach.		Education, Training, Enablement	1) Instruction on how to perform the behaviour; 2) Information about conmsequences; 3) Problem solving	
			Motivation	Automatic	It seems to be habitual for service providers to report that they listen to parents; however this is not clearly reflected in decision making, information provision, and care delivery. Despite good intentions, some service providers stick to their own agenda.	"...I think it's important that we're listening to families but there has to be a purpose for that doesn't it? There's no point listening if we just then merrily go on our way...You're being listened to and what you're saying is then having an influence on the practitioner..." – OSH4. "...I think that is a really sticky point for me because... when a child comes to you and the parent says [what they want to achieve]...OK great that is patient centred and I will obviously make sure that I try to do that...but realistically I am going to do the same thing I was going to do anyway, because I know and have that knowledge...so that feels wrong in my head...because I know we are trying to be really patient centred..." – OSH3	Persuasion, Incentivisation, Coercion, Training, Environmental restructuring, Modelling, Enablement.	1) Feedback on behaviour; 2) Credible source; 3) Prompts and cues; 4) Habit formation; 5) Restructuring the physical environment.	
				Reflective	Service providers have good intentions to listen to parents and validate their concerns; however this seems to be outweighed by pressures associated with routine provision, care pathways and standardised assessment. There is also the feeling that this way of working takes too long and there is already insufficient time. This too needs to be reframed.		Education, Persuasion, Incentivisation, Coercion, Modelling.	1) Behavioural experiments; 2) Feedback on behaviour.	
			Opportunities	Social	There is good intention from service providers to listen and validate parent carer concerns. This is also encouraged by professional and organisational stakeholders.				
				Physical	Service providers need additional time to give space for parents to share their concerns.		Training, Restriction, Environmental Restructuring, Enablement.	1) Prompts and cues; 2) Instruction on how to perform the behaviour.	
7	Adopt a personable approach towards parent carers	Service Providers	Capabilities	Physical					Education/Training: Content should include: 1) Information about the impact of more 'personable' behaviours and how this is prioritised in the therapeutic relationship by parent carers; 2) Information about how to be more personable, without compromising professional responsibility; 3) Examples of some small changes to practice that can improve parent carer perception of personable care; 4) Information about personalised care vs personable care. *Strategies to enhance impact of training* should include: 1) Using stories and testimony from parent carers (as credible sources of information); 2) Use of posters in the clinic room to remind health professionals of desired behaviour. Practice Changes: 1) Introduce sharing of information prior to consultation (what to expect, who they will see, quetsions to think about, etc). This needs to be offered in a personal way e.g. video message questions. Monitoring/Maintaining behaviour: Encouragement of structured supervision and reflection on performance of 'personable' behaviours and actions; soliciting feedback from parent carers.
				Psychological	Service providers need to know how to take a personable approach, whilst maintaining their professional position (operationalise the therapeutic relationship). Service providers also need to know the added perceived value of taking a personable approach (being warm, approachable, kind, considerate, etc) and know how to prioritise this in their clinical contacts.	"She listened, and she showed emotion...and she got where I was coming from. She got the impact his condition was having on me and him, and our wider family, she got it, she understood it, and she empathised. She was real, she was human..." – P10	Education, Training, Enablement	1) Information about environmental and social consequences; 2) Information about health consequences; 3) Problem solving; 4) Feedback on Behaviour	
			Motivation	Automatic	While parents are keen to have more personal contact with health professionals, service providers themselves are habitually more guarded.	"...For example, one of the parents asked me to be friends with her on Facebook. I thought I would just ignore it, but I knew that when I saw her next she would ask me about it. She's very kind of... she likes me, it's so clear [laughs] I'm not boasting, she likes me because I've known her child from birth. She treats me as part of her family, when I go home she tries to treat me like a family member, a cup of tea, or juice, I decline everything. It's not declined, you know what I mean? I try to be professional." - OSH8	Persuasion, Incentivisation, Coercion, Training, Environmental restructuring, Modelling, Enablement.	1) Incompatible beliefs; 2) Framing/reframing.	
				Reflective	Most service providers report recognising the benefits of taking a more personable approach, but hold reservations about how this might be done. Clinician not friend.	" I think the relationship does change, and I don't always necessarily know if that's helpful as well. It's really nice that people can feel like they relate to you in that way, but I think it is helpful for you to still have some boundary. It's helpful on both sides." - OSH2 "...I think the same, being open, friendly, and professionally approachable, and open professionally is different to a friend. I think maintaining a friendship with the patient group is not healthy." - OSH8	Education, Persuasion, Incentivisation, Coercion, Modelling.	1) Problem Solving; 2) Self monitoring;	
			Opportunities	Social	Service providers generally feel that it is important to maintain a professional relationship with parent carers and that care should be taken not to be too close/personal.		Restiction, Environmental Restructuring, Modelling, Enablement.	1) Incompatible beliefs; 2) Framing/reframing.	
				Physical	Some current practice can be depersonalising for parent carers - (e.g. appointment letters are full of different codes and pre-populated data, which can make it difficult for parent carers to know what the appointment is for and who it is with - also what might be the purpose of the appointment).		Training, Restriction, Environmental Restructuring, Enablement.	1) Instruction how to perform the behaviour	
