# Supplementary material for: Empowering Parent Carers of Children With Neurodisability in Healthcare Settings: Co‐Producing a Theory of Change for Intervention Development
Source: Health Expect. 2026 Sep 9;29(5):e70871. doi: 10.1111/hex.70871 (PMC13556309; doi:10.1111/hex.70871)
Supplement: Supplementary file 2 [file HEX-29-e70871-s001.docx]

### Supplementary Material 1: Eligibility Criteria

|  | Parent Participants | Other Stakeholder Participants |
| --- | --- | --- |
| Inclusion Criteria | Any parent carer of a child (aged 0-19 years) accessing children’s services from the hosting NHS trust. | Any health professional or support staff working directly with children and their families in the hosting NHS trust.  Or  Any person responsible for managing health services for children in the hosting NHS trust.  Or  Any person responsible for commissioning health services for children in the hosting NHS trust. |
| Exclusion Criteria | None | Newly qualified clinical staff (under 12 months clinical) experience. |
