# Supplementary material for: Empowering Parent Carers of Children With Neurodisability in Healthcare Settings: Co‐Producing a Theory of Change for Intervention Development
Source: Health Expect. 2026 Sep 9;29(5):e70871. doi: 10.1111/hex.70871 (PMC13556309; doi:10.1111/hex.70871)
Supplement: Supplementary file 3 [file HEX-29-e70871-s003.docx]

## Supplementary file 3 – TIDieR Checklist

Brief name: **Provide the name or a phrase that describes the intervention.**

Working in Partnership with Parent Carers (WiPPC).

Why: **Describe any rationale, theory, or goal of the elements essential to the intervention.**

Better understanding of existing power dynamics in the therapeutic relationship, individualised sharing of information and a more personable approach, may contribute a more collaborative partnership between health professionals and parent carers and to improved parent carer empowerment

What (material): **Describe any physical or informational materials used in the intervention, including those provided to participants or used in intervention delivery or in training of intervention providers. Provide information on where the materials can be accessed (for example, online appendix, URL).**

Materials used in the intervention include;

- a detailed training plan for health professionals (this will include pre-recorded materials of parent carer stories and of an example consultation).
- A facilitator handbook (to be produced following first round of feasibility testing).
- a partnership agreement proforma.
- a supervision proforma.

What (procedures): **Describe each of the procedures, activities, and/or processes used in the intervention, including any enabling or support activities.**

The intervention includes 3 components:

1) *A training programme for health professionals*, consisting of 2 modules.
   a. Working with parent carers – Module 1: Recognising and addressing imbalances of power.
   b. Working with parent carers – Module 2: Sharing information, working in partnership and developing a trusting relationship.
2) A change to practice, consisting of 3 new initiatives:
   a. A partnership agreement.
   b. Sharing an individualised message before the first consultation.
   c. Sharing summary of information after appointments.
3) Additional structured supervision and peer support.

Box 1 provides more detailed description about each component of the intervention, including specific learning objectives and content of the training component.

Who provided: **For each category of intervention provider (for example, psychologist, nursing assistant), describe their expertise, background and any specific training given.**

*Training component:* In the short term (during testing and refinement), training will be provided/delivered by the research team who have developed the intervention. In the medium term, training content will be pre-recorded allowing the two modules to be facilitated by someone who has previously received the training (qualified health professional). A training support guide will be produced for facilitators to follow.

*Practice change component:* The three initiatives described will be delivered by the health professionals who have received the training. The rationale and strategy for delivering the initiatives will be covered in the training component of the intervention.

*Supervision component:* Supervision will be delivered by a peer support group convened following the training component of the intervention. This will be peer led and run alongside existing supervisory systems.

How (mode of delivery; individual or group): **Describe the modes of delivery (such as face to face or by some other mechanism, such as internet or telephone) of the intervention and whether it was provided individually or in a group.**

*Training component:* This will be delivered face to face (either in person or online) to a group of health professionals. An important part of the training is the facilitated discussion and group work.

*Practice change component:*

a) Partnership agreement – this will be completed in person during the first meeting between the parent carer and health professional.
b) Sharing an individualised message before the first consultation – this will be delivered as a voice note and a photograph of the health professional.
c) Sharing summary of information after the appointment – this will be delivered by the health professional in a format agreed in the partnership agreement.

*Supervision component:* This will be run face to face (either in person or online) as a peer support group.

Where: **Describe the type(s) of location(s) where the intervention occurred, including any necessary infrastructure or relevant features.**

*Training component:* This will be delivered as part of staff training and will be integrated into existing training schedules. If delivered in person, adequate space and presentation equipment will be required. If delivered online, virtual presentation software can be used (this will include a facility to move into small group/breakout rooms).

*Practice change component:*

a) Partnership agreement - This will be delivered in person in the location of the first face to face appointment. Typically this would be in the clinic; however it can also be completed during a home visit.
b) Sharing an individualised message before the first consultation - N/A.
c) Sharing summary of information after the appointment – N/A.

*Supervision component:* This will run as part of existing staff supervision infrastructure. Typically this is run face to face (online); however it may also be run in person. If it is run in person, adequate space will be required.

When and how much: **Describe the number of times the intervention was delivered and over what period of time including the number of sessions, their schedule, and their duration, intensity or dose.**

*Training component:* For each delegate, training content will be delivered once in the form of two x 2 hour sessions. These sessions could be delivered separately on different days, or together as part of a longer training day.

*Practice change component:*

a) Partnership agreement – The partnership agreement will be completed during the first face to face appointment. It is expected to take 15 minutes to complete. The agreement will need to be reviewed and updated intermittently (it is expected that this would be every 4 months, or sooner if needed).
b) Sharing an individualised message before the first consultation – This is a one time message. It is expected to take 5 minutes to record and send.
c) Sharing summary of information after the appointment – This will happen following every appointment. It is expected to take an extra 5 minutes to complete.

*Supervision component:* This will be part of existing staff supervision and will run quarterly for 60mins. It may be that additional sessions will be required initially.

Tailoring: **In tailored interventions, not all participants receive an identical intervention. Was this intervention planned to be personalised, titrated or adapted? Yes**

*Training component:* In the medium term, training content will be prerecorded; however, discussion and critical reflection will be guided by the training facilitator, allowing for some tailoring to the groups’ specific needs.

Practice change component:

a) Partnership agreement – The partnership agreement will be negotiated using a proforma; however this will be tailored to the individual needs of the family, which will be reflected in a unique document.
b) Sharing an individualised message before the first consultation – N/A
c) Sharing summary of information after the appointment – This will be tailored to the individual preference/needs of the parent carers (as recorded in the partnership agreement).

How well (planned): **Fidelity refers to the degree to which an intervention happened in the way the investigators intended it to. This item—and item 12—extends beyond simple receipt of the intervention (such as how many participants were issued with the intervention drug or exercises) and refers to “how well” the intervention was received or delivered (such as how many participants took the drug/did the exercises, how much they took/did, and for how long).**

N/A.

This section will be updated following first rounds of feasibility testing.

## Box 1: Structure of the proposed intervention:

The co-created intervention consists of three interconnected components.

### 1) Staff training: 2 x 2 hour interactive training sessions:

- Working with parent carers – Module 1: Recognising and addressing imbalances of power.

##### Key Learning Objectives:

a) To understand what is meant by an imbalance of power, including noticing how it is created and maintained, in relation to working with parent carers.
b) To explore additional factors that influence the power imbalance.
c) To understand the benefits of addressing the power imbalance.
d) To identify some strategies to address the power imbalance within a clinical relationship.

- Working with parent carers – Module 2: Working in partnership, sharing information and developing a trusting relationship.

##### Key Learning Objectives:

a) To review the concept and theoretical underpinnings of family centred practice.
b) To understand the challenges with offering personalised care.
c) To recognise the value of personable care.
d) To introduce a ‘new way of working’, consisting of three changes to current practice, that have been designed to help empower parent carers.

Training will be delivered live, either in person or online. It will consist of pre-recorded content (including video testimony from parent carers and case study examples), provision of information and guided discussion. All provisional content has been co-created with relevant stakeholders, including parent carer partners.

Module 2 will also present the proposed changes to practice which constitute the second component of the intervention.

### 2) Changes to practice:

There are 3 practice changes included in the proposed intervention.

a) Partnership agreement: A document written collaboratively by the health professional and parent carer, during the child’s first appointment. This will set out the terms and expectations of the therapeutic partnership.

It will include the following:

- A statement about the health professional’s role, and how they might fit into the family’s team.
- An agreement about the support needed by parent carers to be able to make informed decisions about their child or young person’s care.
- A record of how parent carers would like information to be shared
  - Content (e.g. brief bullet points summarising the consultation and any actions, comprehensive report, both etc)
  - Delivery (e.g. Letter, email, WhatsApp, SMS, etc).
- A record of the mechanism parent carers can use to ask questions and offer feedback.
- An agreement that questions/feedback will be responded to by the health professional within a given time scale.
- A record of professional’s contact details and hours of work.
- A record of parent carer’s availability for appointments.
- An agreement about visiting child in nursery/school setting.
- An agreement about when an episode of care will be finished.
- A record of any other reasonable adjustments needed by the family.

b) Individualised message before first consultation: Prior to a child’s first appointment, health professionals will send a photo and a voice message to the parent carer. This will include the following information:
   - A personalised introduction
   - A brief description of what the appointment is for
   - A brief description of what to expect when you arrive
   - Questions the family might like to consider
c) Sharing a summary of information after the appointment: (as documented in the partnership agreement). This would include the following:
   - A summary of the consultation (brief bullet points/key takeaway message), shared in the way requested by the parent carers, within an agreed time (ideally within 24 hours).
   - This should include clear indication of when/how parent carers can ask questions, offer feedback and respond.
   - A clear plan for how the health professional will answer any questions raised, within agreed time frame.

### 3) Ongoing structured supervision and support for staff:

This will be linked to existing supervision processes within the organisation. There will be time allocated to discuss how changes to practice are being managed, specifically how staff are responding to feedback and adapting to families’ individual needs.
